# Association study of polymorphisms in synaptic vesicle-associated genes, *SYN2 *and *CPLX2*, with schizophrenia

**DOI:** 10.1186/1744-9081-1-15

**Published:** 2005-08-31

**Authors:** Hee Jae Lee, Ji Young Song, Jong Woo Kim, Sheng-Yu Jin, Mi Suk Hong, Jin Kyoung Park, Joo-Ho Chung, Hiroki Shibata, Yasuyuki Fukumaki

**Affiliations:** 1 Medical Science Institute, Kangwon National University, Chunchon, Republic of Korea; 2 Department of Neuropsychiatry, College of Medicine, Kyung Hee University, Seoul, Republic of Korea; 3 Kohwang Medical Research Institute, College of Medicine, Kyung Hee University, Seoul, Republic of Korea; 4 Division of Disease Genes, Research Center for Genetic Information, Medical Institute of Bioregulation, Kyushu University, Fukuoka, Japan

## Abstract

**Background:**

The occurrence of aberrant functional connectivity in the neuronal circuit is one of the integrative theories of the etiology of schizophrenia. Previous studies have reported that the protein and mRNA levels of the synapsin 2 (*SYN2*) and complexin 2 (*CPLX2*) genes were decreased in patients with schizophrenia. Synapsin 2 and complexin 2 are involved in synaptogenesis and the modulation of neurotransmitter release. This report presents a study of the association of polymorphisms of *SYN2 *and *CPLX2 *with schizophrenia in the Korean population.

**Methods:**

Six single nucleotide polymorphisms (SNPs) and one 5-bp insertion/deletion in *SYN2 *and five SNPs in *CPLX2 *were genotyped in 154 Korean patients with schizophrenia and 133 control patients using direct sequencing or restriction fragment length polymorphism analysis. An intermarker linkage disequilibrium map was constructed for each gene.

**Results:**

Although there was no significant difference in the genotypic distributions and allelic frequencies of either *SYN2 *or *CPLX2 *polymorphisms between the schizophrenia and control groups, the two-way haplotype analyses revealed significant associations with the disease (*P *< 0.05 after Bonferroni correction). The three-way haplotype analyses also revealed a significant association of *SYN2 *with schizophrenia (*P *< 0.001 after Bonferroni correction).

**Conclusion:**

These results suggest that both *SYN2 *and *CPLX2 *may confer susceptibility to schizophrenia in the Korean population.

## Background

Schizophrenia is a severe, chronic mental illness affecting 0.5–1.5% of the general population worldwide [1]. The contribution of genetic factors to the vulnerability to schizophrenia has been well established by family, twin, and adoption studies that have suggested a significant heritability of approximately 50–70% [2]. Many studies have attempted to identify the allelic variants that confer susceptibility to the illness, but no single genes have been identified that produce a major effect on the vulnerability [3].

Recently the synaptic hypothesis of schizophrenia has gained attention by attributing the fundamental pathology of schizophrenia to the dysfunction of synaptic transmission involving various molecules [4]. Synapsins, a family of synaptic vesicle-associated phosphoproteins, play a crucial role in the regulation of neurotransmission, synaptogenesis, and neuronal plasticity [5]. Three human synapsin genes have been identified (*SYN1*, *2*, and *3*; OMIM 313440, 600755, and 602705) [6]. Complexin 1 and complexin 2, which are encoded by *CPLX1 *(OMIM 605032) and *CPLX2 *(OMIM 605033), respectively, and are also called synaphins, are pre-synaptic membrane proteins that preferentially bind to syntaxin within the SNARE (soluble N-ethylmaleimide-sensitive fusion attachment protein receptors) complex. These proteins are important regulators of transmitter release immediately preceding vesicle fusion [7]. Previous studies have demonstrated that the concentrations of synapsins and complexins are reduced in the brains of schizophrenics [8,9]. The expression levels of both synapsins were significantly decreased in the hippocampal tissue of schizophrenic patients [10]. The levels of synapsin 2 and complexin 2 mRNA were also significantly reduced in the prefrontal cortex, cerebellum, and hippocampus of schizophrenics [11-14].

*SYN2 *was mapped to chromosome 3p25 [15], and *CPLX2 *is located on chromosome 5q35.3 (OMIM 605033). These loci were identified as potential regions conferring susceptibility to schizophrenia in diverse populations [16-18]. Based on their localization, well-established neurobiological roles, and expression patterns in schizophrenic patients, we selected *SYN2 *and *CPLX2 *as candidate genes for conferring susceptibility to schizophrenia. In this report, we present an association study of *SYN2 *and *CPLX2 *with schizophrenia using 12 polymorphisms in the Korean population.

## Results

### *SYN2 *polymorphisms in the schizophrenia and control groups

**Figure 1:**
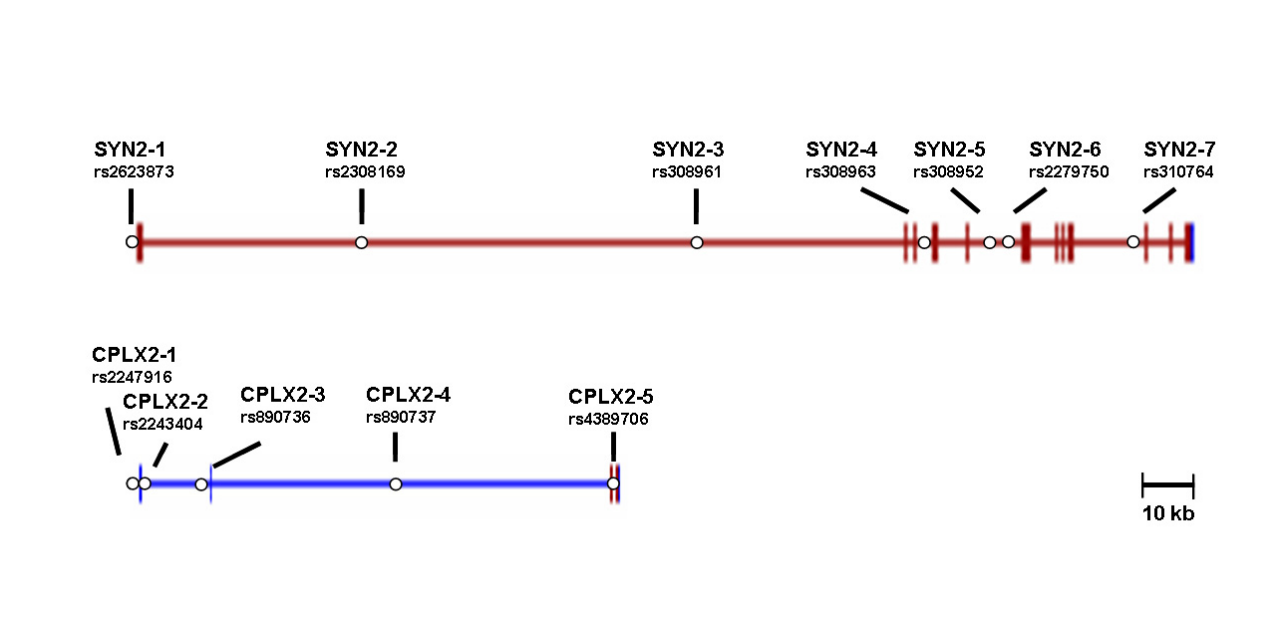
**Genomic organization of *SYN2 *and *CPLX2 *and locations of SNPs**. **a**; *SYN2 *spans over 140 kb and is composed of 14 exons. Seven markers are indicated with the dbSNP reference ID . **b**; *CPLX2 *spans over 83 kb and is composed 3 exons. Five markers are indicated with the dbSNP reference ID .

Of the seven polymorphisms in *SYN2*, rs2623873 (SYN2-1) is located in the promoter region, whereas the others are all located in the intronic regions (SYN2-2–7) (Fig. 1, Table 1). The genotypic distributions and allelic frequencies of polymorphisms in *SYN2 *were determined in 113 schizophrenic patients and 114 normal healthy controls by direct sequencing or *Dde*I RFLP. The genotypic distributions and allelic frequencies of polymorphisms in *SYN2 *are shown in Table 2. The average allelic frequency of the SNPs was 0.312. Given the equivalent frequency for the susceptible allele, the expected detection power for *SYN2 *was 0.9538 to 0.9929 under the multiplicative model with a genotype relative risk = 1.8 to 2.0 [22]. None of the SNPs showed any significant deviation from Hardy-Weinberg equilibrium (*P *> 0.05). We observed no significant difference in the genotypic distributions and allelic frequencies between the schizophrenics and control groups (Table 2).

**Table 1:** Twelve polymorphisms genotyped in this study.

Gene	Name	dbSNP rs#	Region	Allele	Methods
*SYN2*	SYN2-1	rs2623873	Promoter	G/T	Direct Sequencing
*SYN2*	SYN2-2	rs2308169	Intron	ATGCT/-	Direct Sequencing
*SYN2*	SYN2-3	rs308961	Intron	T/G	Direct Sequencing
*SYN2*	SYN2-4	rs308963	Intron	C/G	Direct Sequencing
*SYN2*	SYN2-5	rs308952	Intron^a^	A/G	*Dde*I RFLP
*SYN2*	SYN2-6	rs2279750	Intron^a^	A/C	Direct Sequencing
*SYN2*	SYN2-7	rs310762	Intron	C/T	Direct Sequencing

*CPLX2*	CPLX2-1	rs2247916	Promoter	G/T	*Mae*III RFLP
*CPLX2*	CPLX2-2	rs2243404	5'UTR	C/T	*Cac*8I RFLP
*CPLX2*	CPLX2-3	rs890736	Intron	C/T	*Ava*II RFLP
*CPLX2*	CPLX2-4	rs890737	Intron	C/T	Direct Sequencing
*CPLX2*	CPLX2-5	rs4390706	Intron	G/A	Direct Sequencing

^a ^Tissue inhibitor of metalloproteinase 4 (*Timp4*) gene is nested within the intron of *SYN2 *in reverse orientation.

**Table 2:** Genotype distributions and allele frequencies of each polymorphism of the *SYN2 *and *CPLX2 *in the schizophrenia and control groups.

Polymorphism	Subjects	Genotype distribution (frequency)				Allele frequency		
		11	12	22	*P*^*a*^	1	2	*P*^*b*^
SYN2-1	Cases (n = 113)	47 (0.416)	50 (0.442)	16 (0.142)	0.527	0.637	0.363	0.679
	Controls (n = 114)	41 (0.360)	59 (0.518)	14 (0.122)		0.618	0.382	
SYN2-2	Cases (n = 113)	40 (0.354)	55 (0.487)	18 (0.159)	0.758	0.597	0.403	0.438
	Controls (n = 114)	36 (0.316)	56 (0.491)	22 (0.193)		0.561	0.439	
SYN2-3	Cases (n = 113)	83 (0.735)	30 (0.265)	0 (0.000)	0.762	0.867	0.133	0.975
	Controls (n = 114)	85 (0.746)	28 (0.246)	1 (0.009)		0.868	0.132	
SYN2-4	Cases (n = 113)	49 (0.434)	45 (0.398)	19 (0.168)	0.722	0.633	0.367	0.612
	Controls (n = 114)	44 (0.386)	51 (0.447)	19 (0.167)		0.610	0.390	
SYN2-5	Cases (n = 113)	72 (0.637)	35 (0.310)	6 (0.053)	0.973	0.792	0.208	0.869
	Controls (n = 114)	74 (0.649)	34 (0.298)	6 (0.053)		0.798	0.202	
SYN2-6	Cases (n = 113)	67 (0.593)	39 (0.345)	7 (0.062)	0.786	0.765	0.235	0.622
	Controls (n = 114)	63 (0.553)	44 (0.386)	7 (0.061)		0.746	0.254	
SYN2-7	Cases (n = 113)	39 (0.345)	54 (0.478)	20 (0.177)	0.847	0.584	0.416	0.576
	Controls (n = 114)	42 (0.368)	55 (0.482)	17 (0.149)		0.610	0.390	

CPLX2-1	Cases (n = 154)	132 (0.857)	22 (0.143)	0 (0.000)	0.441	0.929	0.071	0.612
	Controls (n = 133)	113 (0.850)	18 (0.135)	2 (0.015)		0.917	0.083	
CPLX2-2	Cases (n = 154)	132 (0.857)	22 (0.143)	0 (0.000)	0.254	0.929	0.071	0.198
	Controls (n = 133)	108 (0.812)	23 (0.173)	2 (0.015)		0.898	0.102	
CPLX2-3	Cases (n = 154)	115 (0.747)	34 (0.221)	5 (0.032)	0.129	0.857	0.143	0.072
	Controls (n = 133)	85 (0.639)	43 (0.323)	5 (0.038)		0.801	0.199	
CPLX2-4	Cases (n = 154)	110 (0.714)	40 (0.260)	4 (0.026)	1.000	0.844	0.156	0.849
	Controls (n = 133)	94 (0.707)	35 (0.263)	4 (0.030)		0.838	0.162	
CPLX2-5	Cases (n = 154)	124 (0.805)	30 (0.195)	0 (0.000)	0.312	0.903	0.097	0.541
	Controls (n = 133)	112 (0.842)	20 (0.150)	1 (0.008)		0.917	0.083	

^a ^Fisher's exact probability tests, case *vs *controls (2 × 3 genotype-based analysis)

^b ^Fisher's exact probability tests, case *vs *controls (2 × 2 allele-based analysis)

We compared the LD for all possible two-way comparisons of the SNPs in the controls (Table 3). The pairwise D' values for the seven SNPs were consistently high, except in one instance (SYN2-2 *vs*. SYN2-6; D' = 0.300, r^2 ^= 0.200). Out of the 21 possible pairs of SNPs, significant haplotype associations with schizophrenia were observed for 4 pairs: SYN2-1 – SYN2-2 (χ^2 ^= 27.58, df = 3, *P *= 4.45 × 10^-6^), SYN2-2 – SYN2-4 (χ^2 ^= 16.46, df = 3, *P *= 9.12 × 10^-4^), SYN2-2 – SYN2-7 (χ^2 ^= 8.08, df = 3, *P *= 0.044), and SYN2-3 – SYN2-4 (χ^2 ^= 10.66, df = 3, *P *= 0.014) (Table 3). Even after the Bonferroni correction (number of haplotypes, n = 21), the associations of the SYN2-1 – SYN2-2 and SYN2-2 – SYN2-4 haplotypes with schizophrenia remained significant (*P*_*corr *_= 9.35 × 10^-5 ^and *P*_*corr *_= 0.019) (Table 3). The T allele-the deletion allele haplotype for the SYN2-1 – SYN2-2 combination and the deletion allele-the G allele haplotype for the SYN2-2 – SYN2-4 combination were observed more frequently in schizophrenia than the controls (Table 4).

**Table 3:** Pairwise linkage disequilibrium and haplotype association of SNPs in *SYN2*.

	**SYN2-1**	**SYN2-2**	**SYN2-3**	**SYN2-4**	**SYN2-5**	**SYN2-6**	**SYN2-7**
**SYN2-1**		0.532	0.776	0.758	0.865	0.516	0.642
		0.222	0.056	0.553	0.306	0.482	0.397
**SYN2-2**	**4.45 × 10^-6 a^**		0.613	0.453	0.734	0.300	0.429
			0.044	0.250	0.174	0.200	0.225
**SYN2-3**	0.246	0.632		1.000	0.997	0.480	1.000
				0.097	0.038	0.433	0.097
**SYN2-4**	0.845	**9.12 × 10^-4 a^**	0.014		1.000	0.480	0.806
					0.395	0.433	0.650
**SYN2-5**	0.896	0.655	0.135	0.133		0.938	0.865
						0.651	0.295
**SYN2-6**	0.116	0.892	0.602	0.602	0.147		0.722
							0.278
**SYN2-7**	0.646	0.044	0.155	0.392	0.404	0.0.72	

Upper diagonal top: D', bottom: r^2 ^in controls; Lower diagonal: *P *value by *χ*^2 ^test (df = 3)

^a ^*P *< 0.05 after Bonferroni correction

**Table 4:** Estimated haplotype frequencies of the SYN2-1 – SYN2-2 and SYN2-2 – SYN2-4 combination on the *SYN2*

**Haplotypes**		**Estimated frequency**	
**SYN2-1**	**SYN2-2**	**Cases**	**Controls**

T	Deletion	0.583	0.461
T	ATGCT	0.054	0.158
G	Deletion	0.014	0.100
G	ATGCT	0.349	0.281

**SYN2-2**	**SYN2-4**	**Cases**	**Controls**

Deletion	G	0.569	0.463
Deletion	C	0.028	0.098
ATGCT	G	0.064	0.146
ATGCT	C	0.339	0.293

We also investigated the association of three-way haplotypes formed by SYN2-1, SYN2-2, and SYN2-4 with schizophrenia. A significant difference in the haplotype frequencies between the schizophrenia and control groups was observed (χ^2 ^= 35.0, df = 7, *P*_*corr *_= 1.1 × 10^-5^). For the combination of SYN2-1, SYN2-2, and SYN2-4, the estimated frequencies of the T-deletion-G haplotype differed between the schizophrenia (0.570) and controls groups (0.440).

### *CPLX2 *polymorphisms in schizophrenia and control groups

Of the five SNPs in *CPLX2*, rs2247916 (CPLX2-1) is located in the promoter region, rs2243404 (CPLX2-2) is located in the 5'UTR, and the others are located in the intronic regions (Fig. 1, Table 1). We determined the genotypic distributions and allelic frequencies of the SNPs in 154 schizophrenic patients and 133 normal healthy controls by direct sequencing or RFLP analysis. The genotypic distributions and allelic frequencies for *CPLX2 *SNPs are shown in Table 2. The average allelic frequency of the SNPs was 0.126. Given the equivalent frequency for the susceptible allele, the expected detection power for *CPLX2 *was 0.7445 to 0.8802 based on the multiplicative model with the genotype relative risk = 1.8 to 2.0 [22]. None of the five SNPs showed any significant deviations from Hardy-Weinberg equilibrium. We observed no significant differences in genotypic distributions or allelic frequencies between the schizophrenia and control groups (Table 2).

We compared LD for all possible two-way comparisons of the SNPs in controls (Table 5). The pairwise D' values for the five SNPs were consistently low, except in one instance (CPLX2-2 *vs*. CPLX2-4; D' = 0.715, r^2 ^= 0.011). Only one pair of SNPs (CPLX2-1 *vs*. CPLX2-2) showed a significant haplotype association with schizophrenia (χ^2 ^= 16.28, df = 3, *P *= 0.0009), even after the Bonferroni correction (n = 10, *P*_*corr *_= 0.009, Table 5). For the combination of CPLX2-1 – CPLX2-2, the G allele-the C allele haplotype was observed more frequently in the schizophrenia group than the control group (Table 6).

**Table 5:** Pairwise linkage disequilibrium and haplotype association of SNPs in *CPLX2*.

	**CPLX2-1**	**CPLX2-2**	**CPLX2-3**	**CPLX2-4**	**CPLX2-5**
**CPLX2-1**		0.412	0.325	0.058	0.008
		0.136	0.300	0.001	0.008
**CPLX2-2**	**9.0 × 10^-4 a^**		0.309	0.715	0.301
			0.042	0.011	0.001
**CPLX2-3**	0.494	0.056		0.027	0.019
				0.015	0.016
**CPLX2-4**	0.994	0.830	0.564		0.143
					0.000
**CPLX2-5**	1.000	0.564	0.650	0.992	

Upper diagonal top: D', bottom: r^2 ^in controls; Lower diagonal: *P *value by *χ*^2 ^test (df = 3)

^a ^*P *< 0.01 after Bonferroni correction

**Table 6:** Estimated haplotype frequencies of the CPLX2-1 – CPLX2-2 combination on the *CPLX2*

**Haplotypes**		**Estimated frequency**	
**CPLX2-1**	**CPLX2-2**	**Cases**	**Controls**

T	C	0.010	0.036
T	T	0.061	0.047
G	C	0.919	0.862
G	T	0.010	0.055

## Discussion

In this study, we observed significant, pairwise haplotype associations with schizophrenia for two pairs of SNPs in *SYN2 *(SYN2-1 – SYN2-2 and SYN2-2 – SYN2-4; *P*_*corr *_= 9.35 × 10^-5 ^and *P*_*corr *_= 0.019, respectively) and one pair of SNPs in *CPLX2 *(CPLX2-1 – CPLX2-2, *P*_*corr *_= 0.009) (Table 3, 5). The three-way haplotype (SYN2-1, SYN2-2, and SYN2-4) also showed a significant association with schizophrenia (*P*_*corr *_= 1.1 × 10^-5^). The SYN2-1 and CPLX2-1 SNPs are located in the respective promoter regions, -98 and -156. SYN2-1 was located within the GC box motif and CPLX2-1 within the C/EBP motif in a database search . The positive haplotype associations seem to be based on an increase of LD in the schizophrenia group compared to the control group because the D' values of the schizophrenia group were higher than those of the controls [(SYN2-1 – SYN2-2, 0.935 *vs*. 0.531 (schizophrenics *vs*. controls)), (SYN2-2 – SYN2-4, 0.750 *vs*. 0.453)] (Table 3). A similar situation was also observed with the positive association of the haplotype in *CPLX2 *with schizophrenia [CPLX2-1 – CPLX2-2, 0.852 *vs*. 0.412 (schizophrenics *vs*. controls)] (Table 5).

Chen *et al*. [23] recently reported an association study of four SNPs in *SYN2 *using Han Chinese samples. They found significant associations of SNP rs795009 and a haplotype constructed by the four SNPs with schizophrenia. Chen *et al*. [23] and our study examined two SNPs (rs2308169 and rs308963) in common, and their genotypic and allelic frequencies were similar in both studies. Although Chen *et al*. [23] did not mention the pairwise haplotype association study that we performed, they did report a significant difference in the overall four-way haplotype frequencies between schizophrenics and controls. Since two independent studies have reported a significant haplotype association of *SYN2 *with schizophrenia, this gene is probably involved in the pathogenesis of schizophrenia.

Several studies have suggested that the decreased expression of synaptic genes is characteristic of schizophrenia. In the hippocampus of schizophrenic patients, several studies have shown a consistent pattern of decreases in presynaptic proteins and their encoding mRNAs, such as synapsin 2, synaptophysin, and synaptosomal-associated protein-25 (SNAP-25) [8-10,24]. Furthermore, a reduction in the synapsin 2 mRNA levels was observed in the prefrontal cortex of schizophrenic patients [14], but controvertible results have also been reported [25]. The altered expression levels of other presynaptic proteins, complexin 1 and complexin 2, have been reported in schizophrenic patients [11-13]. Interestingly, complexin 1 is enriched in axosomatic regions, inhibitor neurons, and their synapses, while complexin 2 is enriched in the axodendritic terminals [9,26]. The differential expression of complexins 1 and 2 implies their involvement in the excitatory synapse in the hippocampus of schizophrenic patients [11]. These observations suggest that abnormal expression of *SYN2 *and *CPLX2 *may cause the vulnerability to schizophrenia by altering neurotransmitter release and neuroplasticity.

## Conclusion

We found significant differences in the haplotype frequencies in both *SYN2 *and *CPLX2 *polymorphisms between schizophrenia and control groups. In addition, the haplotype constructed from three polymorphisms (SYN2-1, SYN2-2, and SYN2-4) showed a significant association with schizophrenia. Our results suggest that both *SYN2 *and *CPLX2 *polymorphisms may contribute susceptibility to schizophrenia in the Korean population.

## Methods

### Subjects

A total of 154 unrelated Korean schizophrenia patients (80 male and 74 female with a mean ± SD age of 43.8 ± 11.4 yr) and 133 unrelated Korean controls (64 male and 69 female; age 50.6 ± 11.7 yr) were recruited. For the *SYN2 *analysis, 113 unrelated Korean schizophrenia patients (60 male and 53 female with a mean ± SD age of 42.2 ± 11.3 yr) and 114 unrelated Korean controls (60 male and 54 female; age 51.7 ± 10.9 yr) were participated. The schizophrenia patients were diagnosed using the Diagnostic and Statistical Manual of Mental Disorders (DSM)-IV criteria. The control subjects were recruited after they had been designated as mentally healthy in a general health check-up program. The average age of the controls exceeded 50 years because we tried to avoid misincorporation of patients with late onset schizophrenia in the control group, while it may produce statistical bias potentially. Written informed consent was obtained from all subjects. This study was approved by the Ethics Committee of Kyung Hee University, Faculty of Medicine. Genomic DNA was extracted from whole blood cells using a NucleoSpin^® ^Blood kit (Macherey-Nagel, Easton, PA).

### SNP Selection and PCR-based Genotyping

Since the genomic sizes of *SYN2 *and *CPLX2 *are about 187 and 89 kb, respectively, we initially intended to select common polymorphisms at intervals of approximately 20–50 kb from the dbSNP . After validating the frequency of each polymorphism in 24 healthy Korean individuals using direct sequencing, we selected seven common polymorphisms from *SYN2 *and five from *CPLX2 *for further analyses (Fig. 1, Table 1). We amplified the fragments containing polymorphisms individually and genotyped DNA samples for each SNP with either PCR-based restriction fragment length polymorphism (RFLP) assays or direct sequencing performed with an ABI PRISM^® ^Dye Terminator Cycle Sequencing kit (Applied Biosystems, Foster City, CA) on an ABI PRISM^® ^3100 DNA sequencer (Applied Biosystems) (Table 1). In case of unclear sequence data, we repeated direct sequencing under various conditions until the genotype was determined correctly.

### Statistics

The deviation of the genotypic frequencies from Hardy-Weinberg equilibrium was examined using the chi-square test (df = 1). Statistical differences in the genotypic distributions and allelic frequencies between the schizophrenia and control groups were examined using the Fisher's exact probability test. We calculated D' and r^2 ^to evaluate the magnitude of linkage disequilibrium (LD) [19]. We estimated haplotype frequencies using the EH program, version 1.14 [20]. The statistical analysis of haplotype association was done as previously described [21]. We applied the Bonferroni correction to multiple testing based on the number of haplotypes. The significance level for all the statistical tests was 0.05.

## Authors' contributions

HJ Lee conceived of the study, carried out sequencing, participated in the interpretation of the data and drafted manuscript, JY Song, JW Kim and JK Park recruited the samples of schizophrenia patients, SY Jin, MS Hong, and J-H Chung recruited the samples of normal control, H Shibata and Y Fukumaki participated in its design, carried out the statistical analyses, and participated in the interpretation of data. All authors read and approved the final manuscript.
